# Intensity-Stabilized Fast-Scanned Direct Absorption Spectroscopy Instrumentation Based on a Distributed Feedback Laser with Detection Sensitivity down to 4 × 10^−6^

**DOI:** 10.3390/s16091544

**Published:** 2016-09-21

**Authors:** Gang Zhao, Wei Tan, Mengyuan Jia, Jiajuan Hou, Weiguang Ma, Lei Dong, Lei Zhang, Xiaoxia Feng, Xuechun Wu, Wangbao Yin, Liantuan Xiao, Ove Axner, Suotang Jia

**Affiliations:** 1 State Key Laboratory of Quantum Optics and Quantum Optics Devices, Institute of Laser Spectroscopy, Shanxi University, Taiyuan 030006, China; zhaogang030006@126.com (G.Z.); tanwei8648@126.com (W.T.); m18234129833@163.com (M.J.); jiajuanhou@163.com (J.H.); donglei@sxu.edu.cn (L.D.); k1226@sxu.edu.cn (L.Z.); ywb65@sxu.edu.cn (W.Y.); xlt@sxu.edu.cn (L.X.); jggp@sxu.edu.cn (S.J.); 2 Collaborative Innovation Center of Extreme Optics, Shanxi University, Taiyuan 030006, China; 3 Department of Physics, Umeå University, Umeå SE-901 87, Sweden; ove.axner@umu.se; 4 Department of Electrical Engineering and Automation, Shanxi Polytechnic College, Taiyuan 030006, China; fengxx0351@163.com; 5 Shanxi Guohui Optoelectronic Technology CO., Ltd., Taiyuan 030006, China; jerry@ghopto.com

**Keywords:** direct absorption spectroscopy, DFB laser, intensity stabilization, fast scanning, relative intensity noise, polarization rotator

## Abstract

A novel, intensity-stabilized, fast-scanned, direct absorption spectroscopy (IS-FS-DAS) instrumentation, based on a distributed feedback (DFB) diode laser, is developed. A fiber-coupled polarization rotator and a fiber-coupled polarizer are used to stabilize the intensity of the laser, which significantly reduces its relative intensity noise (RIN). The influence of white noise is reduced by fast scanning over the spectral feature (at 1 kHz), followed by averaging. By combining these two noise-reducing techniques, it is demonstrated that direct absorption spectroscopy (DAS) can be swiftly performed down to a limit of detection (LOD) (1σ) of 4 × 10^−6^, which opens up a number of new applications.

## 1. Introduction

As a result of the rapid development of our society, modern industry and agriculture are gradually abandoning traditional, extensive and coarse modes of operation of many types of systems and processes and are instead incorporating finer means of control or even exploiting autonomous systems. Gas Detection in general, and trace gas detection in particular, is one of the most critical constituents of the active control processes that govern many systems in our society and is therefore regarded as an important symbol of modernization [1]. Examples of species that are of importance to detect, already in minor abundances, are ammonia in the process of denitration of coal burning boilers, which is used to control the mixture ratio of urea or ammonia [2]; hydrogen sulfide in the catalytic conversion process that converts coal gas to methane, which is used to control the amount of the total sulfur in methane [3]; and CO_2_ in the growth process of crops that is used to optimize the efficiency of photosynthesis [4]. In addition, there are a number of toxic or environmentally hazardous gases that would cause harm to our environment if emitted in large quantities, e.g., by large energy consuming enterprises. Such gases should, preferably, be monitored at the source so that their amounts can be reduced and kept at acceptable levels by regulation processes. Trace gas detection can also be applied to a variety of other fields, e.g., for the diagnosis of human health [5], for chemical analysis [6], and for air monitoring in space stations [7], just to mention a few. Hence, trace gas detection has an important role in our modern society.

Laser spectroscopy techniques have, over the years, demonstrated a number of appealing advantages to trace gas detection, primarily because of their characteristics of in-time and on-line measurement, high sensitivity, and good selectivity. Due to their simple structure and calibration-free property, most field deployable laser gas analyzers are based on direct absorption spectroscopy (DAS). Despite their advantages, these analyzers are plagued by a common limitation; they detect a small decrease of light, on a background of much larger levels of light, in a system that is affected by a significant amount of low-frequency noise, often referred to as 1/*f* noise. This implies that they can only reach modest limits of detection (LODs) [8].

One means to improve on this is to increase the interaction length between the light and the target gas by a multi-pass cell or an external cavity. Regarding the latter, a series of techniques, e.g., cavity ring-down spectroscopy and various other cavity enhancement techniques, have been developed [9,10,11,12]. However, the high sensitivity to alignment and vibration makes these types of techniques unsuitable for applications in harsh environments.

Another way to improve on the LODs is to use a modulation technique to shift the absorption information to the high frequency region where there is less noise. By this, the relative intensity noise (RIN) of the tunable diode laser, which often has a 1/*f^α^* (*α* > 0) behavior and therefore contributes a large proportion of the noise at lower frequencies, can be significantly reduced [13,14,15]. Based on this, techniques such as wavelength modulation spectroscopy (WMS) [16,17,18,19,20] and frequency modulation spectroscopy (FMS) [21,22,23] have been developed. However, assessments of the concentrations of species addressed from a demodulated signal require calibration, often by the use of a standard reference gas, which severely complicates the setup [24,25,26].

It is also possible to reduce low frequency noise, and thereby improve on the detection sensitivity, by the use of balanced detection (BD). This technique, in which the absorption signal is subtracted from a reference signal, can in many cases remove significant parts of the common-mode laser intensity noise that normally deteriorates unmodulated detection systems. In some cases, it has demonstrated impressive LODs, even down to 5 × 10^−6^ [27,28]. However, this method requires that the two light beams used are affected by external noise, e.g., from the system under study, in the same manner and by equal amounts, which, in practice, is not always the case. It is also sensitive to any possible nonuniformity of photodiodes and circuits, which additionally limits its practical use [14].

An alternative and simple way that can significantly improve on the LOD of DAS is the application of fast scanning over a specific spectral feature, followed by averaging. Such an averaging process acts as a low pass filter that can reduce high frequency noise as long as it is predominantly white and not being caused by drifts. It can then improve on the signal to noise ratio (SNR) by *t*^0.5^ (where *t* is the averaging time) [29,30]. The efficiency of this scheme has been demonstrated in many practical applications [31,32]. Despite this, however, DAS performed by diode lasers is still severely affected by the RIN of the lasers, which limits the performance of the technique.

This letter presents a novel methodology that combines an intensity stabilization of the laser, to reduce its RIN with fast scanning of the laser, followed by averaging, to reduce the influence of white noise in the high frequency range, so as to realize intensity-stabilized, fast-scanned, direct absorption spectroscopy (IS-FS-DAS). The laser was stabilized by use of a fiber coupled polarization rotator (*f*-PR) and a fiber coupled polarizer (*f*-Pol), while the wavelength of the laser was scanned at a rate of 1 kHz. By then averaging over 1000 scans, a detection bandwidth of 1 Hz could be achieved. It was found that by this, the detection sensitivity of DAS could be significantly improved, even beyond those of the conventional modulation techniques. The efficiency of our new IS-FS-DAS methodology is demonstrated by a comparison with that of a conventional DAS detection scheme by measurement of acetylene at concentrations of parts per million (ppm) levels.

## 2. Intensity Stabilization

**Figure 1:**
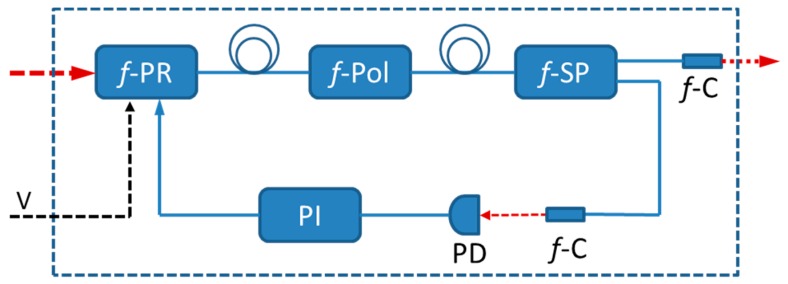
Schematic diagram of the intensity-stabilization of a distributed feedback (DFB) laser; *f*-PR: fiber polarization rotator; *f*-Pol: fiber polarization; *f*-SP: fiber splitter; *f*-C; fiber coupler; PD: photodiode; PI: proportion- integration controller. The *f*-PR and *f*-Pol make jointly up the intensity controller (IC).

The experimental setup for laser intensity stabilization is shown in Figure 1. The intensity controller (IC) consists of a fiber-coupled voltage-controlled polarization rotator (*f*-PR, BATI, PRM) and a fiber-coupled polarizer (*f*-Pol, Connet Fiber Optics Company Limited, Shanghai, China). The key directions of the input/output connectors, which represent the slow axis of the polarization maintaining (PM) fibers, are all aligned. The *f*-PR acts as a half-wave plate and can provide an immediate and continuous rotation of linearly polarized light, thus rotating the included angle between the polarization directions of the light and the *f*-Pol controlled by an applied control voltage. Therefore, by monitoring a part of the output of the IC and using this as a means to control the *f*-PR, the output intensity of the IC can, according to Malus law, be controlled.

To extract the instantaneous laser power, a part of the output of the IC is split off by the use of a fiber splitter (*f*-SP) with a splitting ratio of 1:9. The weak output of the splitter is detected by a photodetector (PD). This signal, subtracted by a preset control voltage (V), is regulated by a PI controller (New Port, LB1005) before it is sent, in the form of an error signal, to the *f*-PR. The strong output of the fiber splitter passes through a gas sample cell and is used as the probe of the gas in the cell.

**Figure 2:**
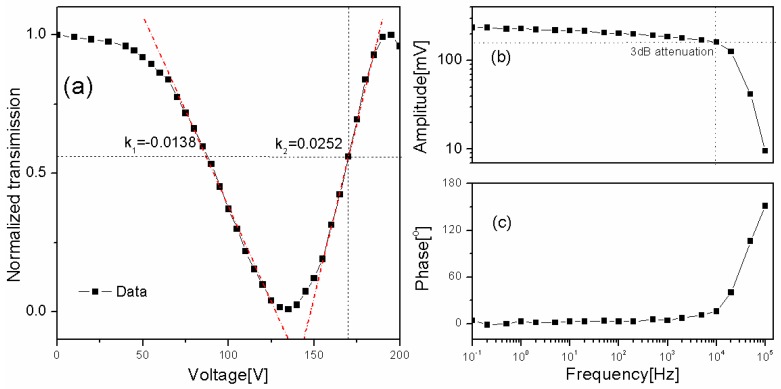
(**a**) Transmission of linearly polarized light through the intensity stabilizer as a function of the voltage applied to the fiber polarization rotator (*f*-PR); Frequency dependence of the (**b**) amplitude and (**c**) phase responses of the PR. Solid squared markers represent individual data points. Blue solid lines make up curves to guide the eye through the individual data points. The blue dotted lines in panel (a) represent the x- and y-values of the nominal working position of the *f*-PR while those in panel (b) indicate the amplitudes and frequency for the 3-dB response. The dashed dotted lines represent the linear responses at the positions for close-to-linear response.

In order to evaluate the performance of the IC for intensity stabilization, Figure 2a shows the output power of the IC (for a constant power of the incoming linearly polarized light) as a function of direct current (DC) voltage sent to the *f*-PR. As can be seen, when the voltage is varied from 0 to 200 V, the output power of the IC shows two maxima and one minimum. This indicates that the polarization direction is rotated more than 180° by the *f*-PR. It is also shown that there are two ranges that show a close-to-a-linear response with respect to the controlled voltage. Due to the non-linear response of the polarization rotator, these two ranges show dissimilar slopes, viz. −0.0138 and 0.0252 (V^−1^), respectively. The second range is chosen for the IC stabilization due to its steeper slope, which gives rise to a faster correction of a given power fluctuation. In order to get an as efficient as possible power correction range for this distributed feedback (DFB) laser, the center bias voltage was therefore chosen as 170 V. 

A series of sinusoidal signals, with a fixed amplitude (1 V) but with different frequencies, ranging from 0.1 Hz to 100 kHz, were then superposed on this DC voltage. Figure 2b shows the frequency response of the *f*-PR, as detected by the PD, in terms of the amplitudes and phase shifts produced by given sinusoidal signals. This graph shows, by the dotted lines in panel b, that the bandwidth is 10 kHz, at which the amplitude is attenuated by 3 dB. It is also shown that the phase shift below 10 kHz is far from 180°, which is suitable for closed-loop and locking applications.

**Figure 3:**
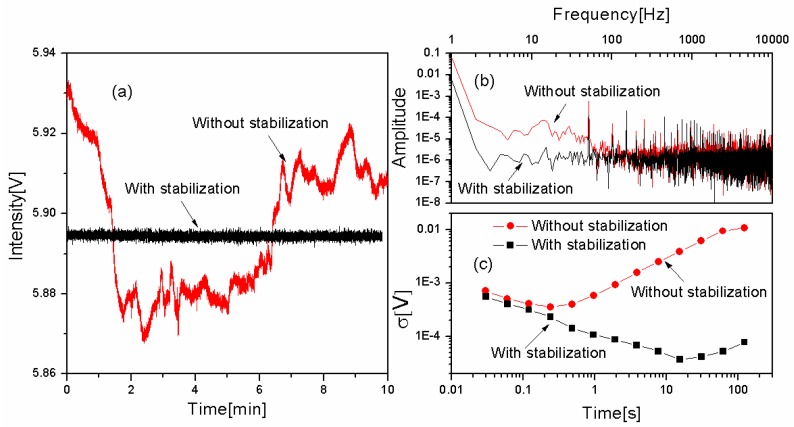
(**a**) Long term monitoring of the light intensity with and without stabilization; the (**b**) corresponding noise spectra and (**c**) Allan-Werle deviations.

A comparison of the intensity of the laser with and without stabilization was then performed. Figure 3a shows a 10 min recording of the light intensity when the DFB laser was allowed to run freely (red in color) and when the intensity was stabilized (black in color). It can be seen that the latter one, whose amplitude was stabilized at 5.895 V, had limited fluctuations, while the intensity of the laser without stabilization, which was found to fluctuate between 5.87 and 5.93 V for the actual measurement period displayed in Figure 3a (and even more for longer observation times), showed significant drifts, resulting from the intrinsic instability of the DFB laser.

In order to assess the improvement of the intensity stabilization, a further analysis was performed. The upper window in Figure 3b and c show a comparison of the noise spectrum with (black in color) and without (red in color) intensity stabilization. It can clearly be seen that the amplitude of the former is much lower than the latter at low frequencies (below 100 Hz). This verifies that the intensity stabilization scheme can decrease the RIN efficiently.

The lower window in Figure 3b shows Allan-Werle plots of two long term monitored signals. For the scheme without intensity stabilization, the Allan deviation (red in color) reaches its minimum (at 3.6 × 10^−4^) rather soon (within a fraction of a second) after which it increases. This indicates that the noise originates predominantly from large drift caused by the RIN rather than white noise. With intensity stabilization, however, (black in color) the Allan deviation is immune to the influence of RIN for integration times slightly beyond 10 s; it reaches its minimum value (3.7 × 10^−5^) for an integration time of 15 s. To both obtain a fast measurement rate and a high sensitivity, an integration time of 1 s was chosen.

## 3. Performance of the System for Detection of Gas

**Figure 4:**
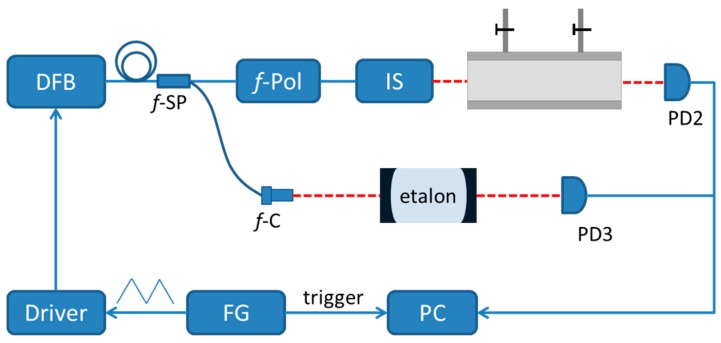
Schematic diagram of the DFB-laser-based intensity-stabilized fast-scanned direct absorption (IS-FS-DAS) spectroscopy instrumentation used in this work. IS: intensity stabilization system; FG: function generation; PC: personal computer.

IS-FS-DAS was then performed using the setup shown in Figure 4. The system was based on a DFB laser (NTT Electronics, NLK1S5GAAA) with a free running line width of 2 MHz, working in the 1527 to 1529 nm range. The wavelength was swept by sending a triangular voltage to the DFB driver (ILX Lightwave, LDC3724C). In order to calibrate the wavelength scale, a 1:9 fiber splitter separated a small part of the output light to a homemade etalon with a FSR (Free spectral range—the frequency spacing between two successive longitudinal modes of high finesses cavity) of 0.022 cm^−1^, while the transmitted light was recorded by a photodiode (PD3, Thorlabs, PDA10CS-EC). The other part of the light was impinging onto a fiber polarizer to form a linearly polarized beam. This light was then sent to the intensity stabilization system described above.

The output light, working as the probe light, was passed through a gas cell with a length of 40 cm and measured by PD2. The gas cell, made of stainless steel, has one gas inlet and one gas outlet. The windows were wedged and made of fused silica. The window of PD2 was slightly tilted to reduce the risk of multiple reflections in the optical system (so called etalons). The data was displayed by an oscilloscope (Tektronix, DPO2024) and acquired by a data acquisition DAQ card (NI, USB6361) with an acquisition rate of 1 MHz and a resolution of 16 bits.

The performance of the system for the detection of gas in trace concentrations was then investigated. The system was scanned at 1 kHz, addressing the ν_1_ + ν_3_ band P_5_(e) transition (at 6544.4419 cm^−1^) of acetylene. The cell was run either with pure nitrogen or 16 ppm acetylene, both at a pressure of 0.92 atm. The concentration of the target gas was obtained by volumetric mixing of a standard reference gas with 428 ppm of acetylene with pure nitrogen using a home build concentration regulation system based on two mass flow controllers (MKS, GM50A). The data were averaged over 1 s (thus averaged over 1000 scans).

**Figure 5:**
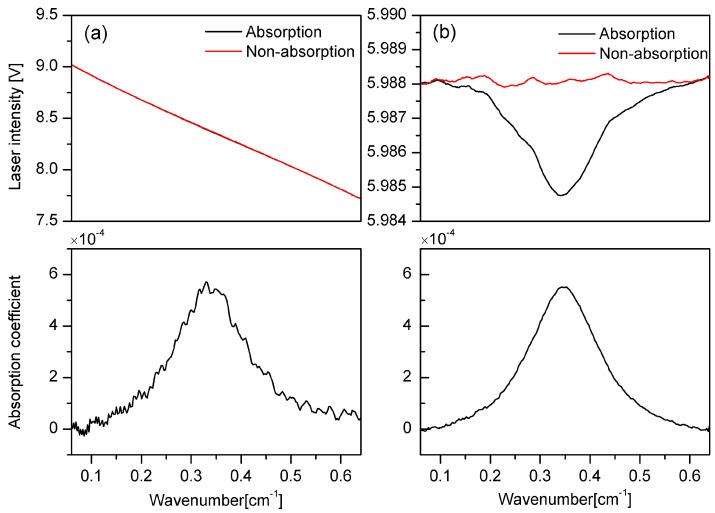
Upper windows: A comparison of the transmitted intensity without (panel **a**) and with (panel **b**) intensity stabilization. The red curves represent measurements from an empty gas cell while the black ones correspond to the case when 16 ppm of C_2_H_2_ is in the cell. Lower windows: The corresponding absorption coefficients created by the use of Beer’s law and the data in the upper windows.

The upper window in Figure 5a shows the average light intensity without intensity stabilization. Because of the intrinsic intensity variation of the DFB laser, the transmitted intensities in the absence (red in color) and in the presence (black in color) of acetylene, are difficult to distinguish from each other. Thus, the absorption feature is drowned in the background. The corresponding absorption coefficient, based on the Beer-Lambert law and shown in the lower window of Figure 5a, shows significant fluctuations, which originate from the RIN of the laser. The profile is also asymmetric, which is caused by the associated intensity modulation.

Figure 5b shows the result with intensity stabilization. Although the intensity amplitude is smaller than that of Figure 5a, which is caused by the fact that the set point of the intensity stabilization must be below the minimum of the intensity amplitude, the light intensity in Figure 5b is much more stable and the absorption feature can be recognized directly from the measured intensity, even under such weak absorption as that from 16 ppm of C_2_H_2_. The absorption coefficient, derived from the novel IS-FS-DAS system presented in this work, illustrated in the lower window, shows a larger signal-to-noise ratio (SNR) and therefore a better LOD than that of the conventional system. The structure of the intensity from an empty cavity in the upper window of Figure 5b arises from the interference between various optical components (referred to as etalon signals), which repeatedly has been shown to be difficult to completely eliminate.

The tuning range of the laser was presently restricted to 0.7 cm^−1^, which, as can be seen from the figure, is sufficient to cover an individual spectral absorption feature. If a wider range is needed, an improved design of the servo loop, to increase the gain in low frequency region, for example, using a cascaded-integrators approach, needs be implemented [33].

**Figure 6:**
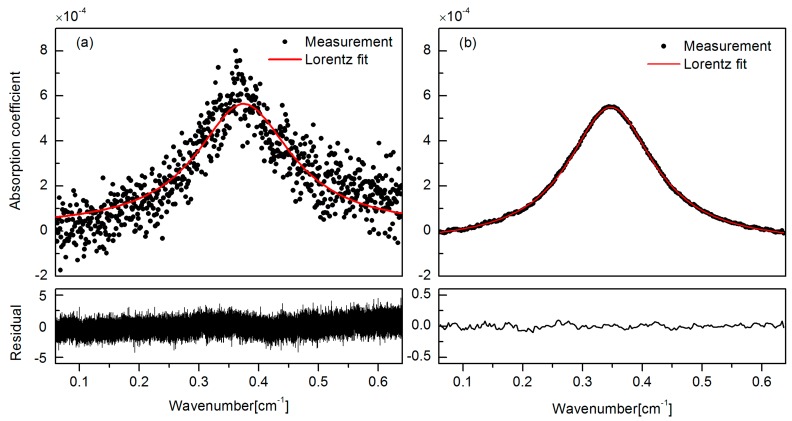
A comparison of absorption coefficients obtained by the use of the (**a**) conventional and (**b**) new DAS scheme for a given measurement time (1 s).

A comparison of the conventional DAS and the new IS-FS-DAS scheme was then performed. A 1 Hz triangle scan without intensity stabilization and a 1 kHz one with intensity stabilization were used to probe the same concentration of acetylene (16 ppm). The data obtained by the latter were exposed to an averaging over 1000 measurements, implying that the two acquisitions represent the same detection bandwidth, viz. 1 Hz. The data points in black in the upper window of Figure 6a represent an absorption coefficient from a single scan of the conventional DAS instrumentation while the curve in red represents a fit of the expected Lorentz line shape function by the least-square fitting method. The residual is presented in the lower window. It can easily be seen that the result of the conventional scheme, under the influence of white noise and RIN, leads to poor quality data.

Figure 6b shows the result based on our novel IS-FS-DAS scheme. Note the differences in scales for the residuals between the two panels. It can clearly be seen that the intensity stabilization can suppress the RIN and thereby the fluctuations at low frequencies, while the fast scanning scheme, followed by averaging, can restrict the influence of the white noise, thus resulting in a less noisy, smoother curve. The SNR is improved by 25 times and a LOD of the IS-FS-DAS system (defined as the signal that equals one standard deviation, i.e., 1σ) of 4 × 10^−6^ was obtained.

By combining two noise-reducing methods, i.e., intensity stabilization and fast scanning, under the same condition (e.g., same integration time), the LOD of our system is reasonably better than that of other systems when only the fast laser scanning was used [34]. Benefiting from the intensity stabilization, it is even found to be similar to that of improved WMS setups, in which systematic care has been taken to eliminate source and detector noise and optical etalons from the system, which typically is in the 10^−6^ range [35].

Although not often demonstrated, due to modulation at a higher frequency where there is less technical noise, FMS can potentially obtain a LOD that approximates the shot noise limit (normally ~10^−7^) [29]. It is also possible for our system to reach these levels if a higher scan frequency is used, although this will require a redesign of the servo to the intensity stabilization with a wider bandwidth and a larger gain. For our system, with the detection bandwidth Δ*f* of 1 Hz, a detector current responsivity *η_c_* of 1.05 A/W, and an incident power *P*_0_ of 1 mW, the shot-noise-limited absorption can be calculated by

(1) $${\left(\alpha \right)}_{\min}^{DAS}=\sqrt{\frac{2e\Delta f}{\eta_{c}P_{0}}}=1.6\times 10^{-8},$$

where *e* is the electronic charge. This shows that the result presented in this work is almost 2 orders of magnitude above the shot-noise-limit, which, in turn, shows that there is still room for improvement.

## 4. Conclusions

In conclusion, a novel gas concentration analyzer based on intensity-stabilized, fast-scanned, direct absorption spectroscopy has been developed and scrutinized. A combination of a fiber-coupled polarization rotator and a polarizer has been used to stabilize the intensity and thereby suppress the RIN of the DFB laser. The added instrumentation, which mainly consists of a fiber-coupled voltage-controlled polarization rotator is as small in size as a coin, thus convenient for system integration. A comparison with a non-stabilization scheme shows that the performance of the system is excellent; in comparison with traditional direct absorption spectroscopy without intensity stabilization, an improvement factor of 25 times of the SNR has been obtained. More specifically, it has been demonstrated that DAS with intensity stabilization and a scan rate of 1 kHz, followed by 1 s averaging to decrease the influence of white noise, is capable of producing an astonishing LOD of 4 × 10^−6^ (1σ). This opens up a series of new implementations of DFB-laser-based DAS instrumentation, not least for detection of a variety of toxic and environmentally important species in low concentrations.
